# Assessing the Accuracy of the Association of Coloproctology of Great Britain and Ireland’s Risk Assessment Tool for Polyp Cancer Management: A Retrospective Study

**DOI:** 10.7759/cureus.114139

**Published:** 2026-08-07

**Authors:** Farhan Akram, Sally Maryosh, Omar Kiwan, Khurram Siddique

**Affiliations:** 1 General and Colorectal Surgery, The Royal Oldham Hospital, Northern Care Alliance NHS Foundation Trust, Manchester, GBR; 2 Surgery, Faculty of Biology, Medicine and Health, University of Manchester, Manchester, GBR

**Keywords:** colonic polyps, colorectal cancer surgery, colorectal neoplasia, git endoscopy, malignant polyps, risk assessment tools

## Abstract

Objective

This study aims to evaluate the accuracy of the Association of Coloproctology of Great Britain and Ireland's (ACPGBI) 2013 risk assessment tool in predicting residual disease and lymph node involvement in patients diagnosed with malignant polyps. Other factors such as polyp size, site, and excision type were also evaluated to determine if they increase the risk of residual disease.

Materials and methods

A retrospective analysis of prospectively collected data was conducted in a UK district general hospital from April 2020 to March 2024. Patients with a histological diagnosis of malignant colorectal polyp(s) were included and followed up with endoscopy and CT at the one-year follow-up. Multivariable analysis was used to evaluate the additional risk factors.

Results

Malignant polyps were identified in 125 patients. Ninety-five patients had adenocarcinoma-containing polyps. No patient with a risk of less than 20% had residual malignancy. Of the patients with an ACPGBI risk score of 20% or more (n = 65), 34 underwent surgical intervention. Seven were found to have residual disease, and four had positive lymph nodes in the resected specimen. Total residual disease risk for patients with an ACPGBI risk score of four or more was 18%. Polyp size was the only additional factor found to be a significant independent risk factor for determining the risk of residual disease following endoscopic polyp removal (OR 1.1, Wald X2 15.4, p = 0.007).

Conclusion

The ACPGBI risk tool accurately reflects the risk of residual disease for those within the highest risk category (score of four or more). Polyp size was found to carry a statistically significant risk of predicting residual disease.

## Introduction

Colorectal cancers are one of the leading causes of cancer-related deaths [1]. Over 95% of colorectal cancer cases arise from adenomatous polyps [2,3]. The Association of Coloproctology of Great Britain and Ireland (ACPGBI) defines a 'malignant polyp' as an "adenoma that appears benign macroscopically but in which there is invasion through the muscularis mucosae into the submucosae” and therefore constitutes “an early carcinoma” [4]. Early detection and excision of adenomatous lesions is recommended to prevent progression to invasive cancer. Polyp identification has increased in frequency since the implementation of the national Bowel Cancer Screening Program (BCSP) [5]. Improvements in endoscopic evaluations [6] and advanced imaging techniques such as computerised tomography colonography (CTC) and capsule colonoscopy have also aided in the detection and assessment of polyps [7], enabling earlier detection and more effective treatment plans.

Malignant colorectal polyps pose a management challenge, as the presence of residual neoplasia and lymph node involvement cannot be completely excluded [4]. In 2013, ACPGBI published a risk stratification tool for malignant colorectal polyps based on the comprehensive literature review of the evidence at the time [4]. These recommendations form the basis for decision-making in colorectal multidisciplinary team (MDT) meetings. This tool was last updated in 2013 [4]; as such, there are concerns that it is outdated, as it does not account for many variables in polyp morphology and histology that can influence risk assessments [8].

The lack of recent, robust evidence further complicates decision-making for malignant polyps, creating challenges for MDTs in determining the best management approach for their patients. Fresh data and research are necessary to improve risk stratification and treatment recommendations for clinicians. A thorough assessment of malignant polyp management at a district general hospital was conducted to assess the effectiveness of the 2013 ACPGBI risk stratification tool.

The primary aim of this study was to measure the accuracy of the ACPGBI risk assessment tool in predicting residual disease and lymph node involvement. Secondary objectives included examination of other potential risk factors not included in the ACPGBI risk score that may assist in predicting residual disease. 

## Materials and methods

A retrospective study on prospectively collected data was conducted in a district general hospital in the UK from April 2020 to March 2024. The study was approved by the Northern Care Alliance NHS Foundation Trust (approval no. 25HIP119).

All patients above the age of 18 who had a histological diagnosis of a malignant polyp, a clear management plan formulated through the colorectal MDT meeting, and at least one repeat endoscopy and CT to assess residual disease and complications were included. Residual disease was defined as the presence of luminal malignancy, polypoid regrowth, or positive lymph nodes at the previous polyp removal site within the resected specimen, repeat endoscopy, and/or follow-up CT. Follow-up for at least one year was incorporated into the inclusion criteria. Patients presenting with neuroendocrine tumors (NETs) or recurrent or metastatic disease at initial presentation, as well as patients who did not have a defined management strategy or follow-up, were excluded.

Patient demographics and polyp features (including size, ACPGBI risk score, excision type, and margins) were recorded. Excision type was defined as endoscopic mucosal resection (EMR) or endoscopic submucosal dissection (ESD). Electronic medical records were used to examine the MDT discussions, clinical consultations, rationale of decisions, and the management outcomes. Patients who met the inclusion criteria were then divided into subgroups according to their ACPGBI risk score. 

Binary logistic regression analysis was performed using SPSS Statistics version 23 (IBM Corp., Armonk, NY, USA) to find risk factors that would predict the likelihood of patients remaining with residual disease after polypectomy. The data were found to be normally distributed. Variables with a p-value <0.1 were considered potentially significant. A multivariable binary regression analysis using Wald numbers (chi-squared) was then performed on the potentially significant variables to determine their p-value. Variables identified as having a p-value <0.05 were considered statistically significant and presented as median (IQR) within each ACPGBI risk group. 

## Results

**Figure 1:**
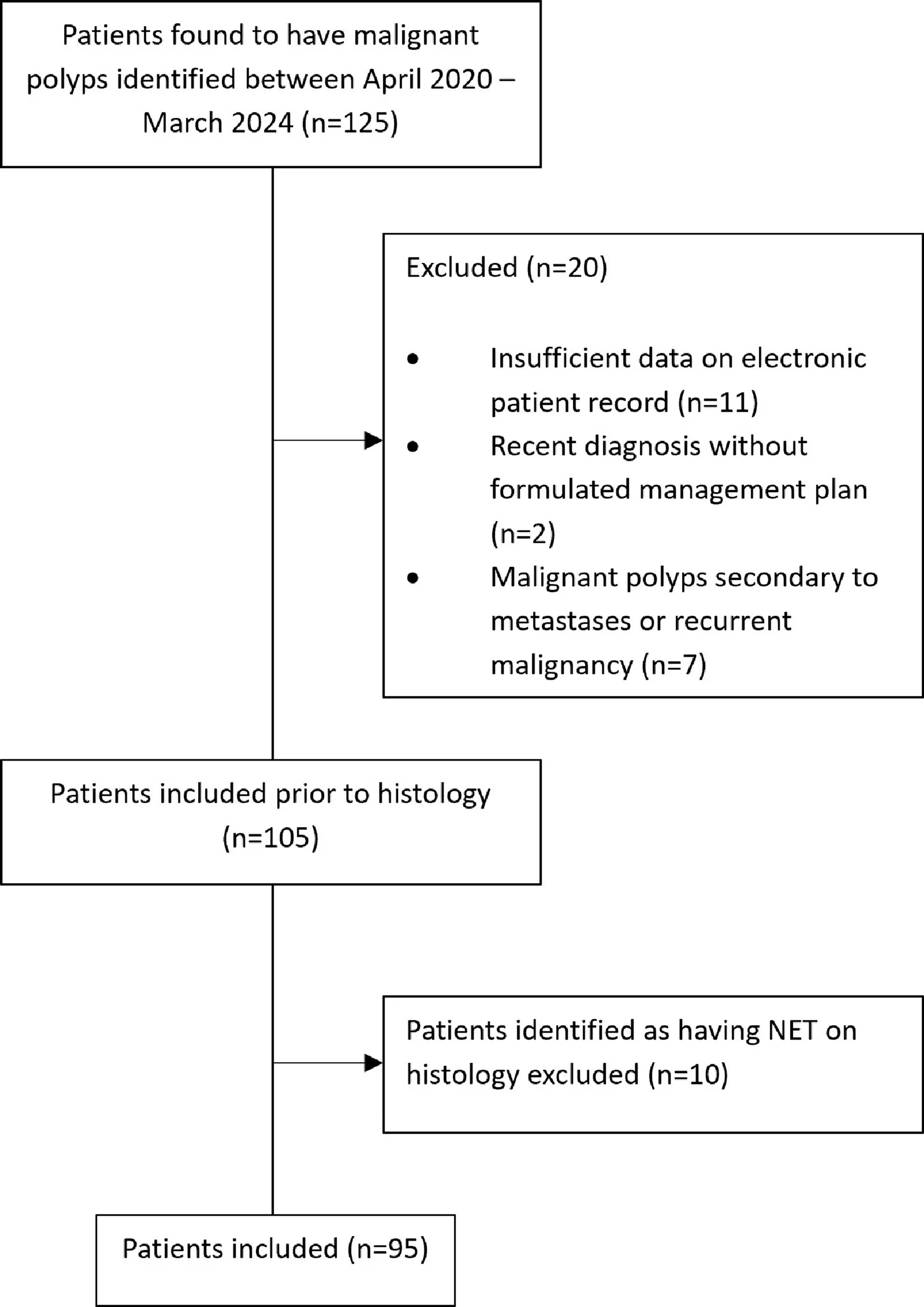
Consolidated Standards of Reporting Trials (CONSORT) diagram of patient inclusion and exclusion

A total of 125 patients were found to have malignant polyps. Of these, 20 patients were excluded; 11 patients had insufficient data on electronic patient records, two were only recently diagnosed without a formulated management plan, and seven had malignant polyps secondary to metastatic or recurrent malignancy. The 10 patients found to have NET on histology were also excluded. The remaining 95 patients were included in the study (Figure 1).

The median age was 68 (range: 31 - 89 years) with a male-to-female ratio of 2:1. Median polyp size was 20 mm (IQR 19). A third (30 patients) were found to have malignant polyps in the rectum. Sixty patients had malignant polyps in the colon (caecum to rectosigmoid junction) and six in the rectosigmoid junction (RSJ). 

Of the 95 patients, 91 (96%) included in the study underwent endoscopic polypectomy; four 4%) who had malignant polyps within the rectum underwent trans-anal resection of tumor (TART) via surgical examination under anesthesia (EUA); 59 (62%) underwent a watch-and-wait approach, while the remaining 36 (38%) underwent surgery.

ACPGBI risk categories

Twelve patients had an ACPGBI risk score <3%, placing them in the zero-risk category. All these patients underwent surveillance. No residual disease was found in any patient. Median polyp size for this group was 20mm (IQR 10).

Seventeen of the 95 patients (18%) had an ACPGBI risk of less than 5%, placing them in risk category 1. Fifteen patients underwent surveillance; two patients underwent surgery (one pan proctocolectomy and one transanal endoscopic microsurgery). One of the two patients had a background of ulcerative colitis; therefore, they underwent a pan-proctocolectomy. No residual disease was found in this group. Median polyp size in this group was 21.5mm (IQR 7.75).

None of the patients included in the study had an ACPGBI risk score of 5% to 10%. Thus, none of the 95 patients were placed in risk category 2. As for risk category 3, only one patient had an ACPGBI risk of 8% to 15%. This patient opted for a watch-and-wait approach with no residual disease found at one year. The polyp size was 12 mm.

Sixty-five patients (68%) had an ACPGBI risk score >20% and were placed in the subgroup of risk category 4. Of these 65 patients, 31 underwent surveillance while 34 underwent further surgical intervention. Overall, 12 patients had residual disease, equating to 18% of patients within this category. Of the 34 patients who underwent surgical intervention, seven were found to have residual disease, and four showed lymph node involvement within the resected specimen. Within the surveillance group, 10 were deemed unfit for surgical intervention at the time of diagnosis, and 21 were offered operative intervention but chose non-operative surveillance. One patient in the surveillance group was found to have residual disease, which unfortunately was found to be metastatic disease at the one-year follow-up. This patient was deemed unfit for surgical intervention at the time of diagnosis due to complicating co-morbidities. The median polyp size was 27.5 mm (IQR 15) in those found to have residual disease, including the patient who did not have surgical intervention.

Correlation of ACPGBI risk and presence of residual disease

Residual disease was only identified within the highest risk category of the ACPGBI risk tool (Score 4+: risk >20%). This suggests that the risk tool is accurate in predicting when residual disease is likely following polypectomy and therefore can be safely used to guide management options during MDT. Only patients within the 'high risk' ACPGBI risk category (4 or more/>20% risk) were subsequently identified as having residual disease at one year, as outlined in Table 1.

**Table 1:** The number of patients within each ACPGBI risk stratification category and within each group who were managed conservatively vs. operatively and identified to have residual disease, stratified per the management they received 1mm = millimeters 2 IQR = interquartile range; ACPGBI: Association of Coloproctology of Great Britain and Ireland

ACPGBI risk category		0	1	2	3	≥4
ACPGBI percentage risk, %		<3	<5	5-10	8-15	>20
Number of patients, n		12	17	0	1	65
Management, n	Surveillance	12	15	-	1	31
	Resection	0	2	-	0	34
Residual Disease, n	Surveillance	0	0	-	0	1
	Resection	-	0	-	-	11
Median polyp size, mm^1^ (IQR)^2^		20 (10)	21.5 (7.75)	-	12	27.5 (15)

Examination of other risk factors, not including ACPGBI risk score

Following binary linear regression analysis of the excision type, both polyp size and polyp site were found to be potentially significant risk factors (p <0.1) in the prediction of residual disease (Table 2). Wald statistics were then used to calculate the p-values of these risk factors. Only polyp size returned a value of statistical significance (p <0.05). For excision type, effect estimates and corresponding Wald statistics were not estimable due to sparse data and an unstable regression coefficient. Multivariable analysis found only polyp size to be significant (Table 2). The polyp site was divided into the anal canal, rectum, RSJ, and colon (caecum to colon). 

**Table 2:** Results of a multivariable binary regression analysis The p-values were found using the respective Wald statistics.

Variable	OR (95% CI)	Wald χ²	p-value
Excision type	Not estimable	n/a	0.999
Polyp size, mm	1.1 (1.0 to 1.1)	15.4	0.007
Site	1.2 (0.5 to 2.6)	0.19	0.701

Polyp size was seen to have an association with the presence of residual disease. None of the cases with a polyp size less than 10 mm showed residual disease compared to 8.8% with a size of 10 to 20 mm, 18.8% of those with a size of 21 to 30, 33.3% with a polyp size of 31 to 40 mm, and half of the patients with a polyp size greater than 40 mm.

## Discussion

This study shows the ACPGBI risk scoring tool is reasonably accurate in predicting the likelihood of residual disease in patients falling within the 'very high' grade of risk (ACPGBI risk score of 4 or more). Korambayil et al. [9] also demonstrated that an ACPGBI risk score of 4 carried a risk of residual disease and lymph node positivity in resection specimens of patients who underwent operative management following en-bloc resection of a malignant polyp. Unlike the current study, Korambayil et al. [9] did not provide specific analysis of each ACPGBI risk category. With minimal literature evaluating the ACPGBI risk scoring tool, it is reassuring to note that this study and comparative literature support the ACPGBI risk score recommendations that patients scoring 4 or more should have operative management discussed and offered, where appropriate. Although this is valuable information, it is important to note that this study has limitations, including being single-center, having a small sample population, and being conducted over a short follow-up period.

The ACPGBI approximates patients within the 'very high' risk category as having a 20% risk of residual disease. The authors of this current study found it to be 18% (12 out of 65) in this single-center population. Korambayil et al. report 14.2% [9], while another comparative paper, Malik and Williams [10], quote that only four of 52 patients had a presence of residual disease, giving a percentage risk of only 8.3%. Our study matches the suggested risk of residual disease and lymph node positivity as described by the ACPGBI. The discrepancy in risk of residual disease in these high-risk groups may be due to our study having the larger, albeit small, sample size between the three papers.

This study did not reveal the presence of any residual disease in patients who had an ACPGBI risk score between 0 and 3. Our findings support the recommendation of non-operative management in patients falling in the lower-risk categories (ACPGBI risk score 0-1). One patient in the 'low risk' (ACPGBI risk score 1) category did undergo surgical resection following the identification of a malignant polyp, but, as mentioned in the ACPGBI risk score, other factors contributed to making this decision, such as an underlying diagnosis of inflammatory bowel disease. Such patients require personalized management outside of the ACPGBI risk tool recommendations, truly requiring an MDT discussion to determine the best management option.

More studies with a larger number of patients and over a period longer than one year will need to be completed to truly investigate an estimated potential risk of residual cancer in all patients. This is especially true for those within the 'medium' and 'high' risk categories (ACPGBI risk scores 2 and 3, respectively). The ACPGBI risk score recommends “discussion or serious consideration of operative management” [4] for patients who fall within ACPGBI risk scores of 2 or 3 where ACPGBI predicts a 5% to 15% risk of residual cancer. Neither this study nor others [9,10] have demonstrated evidence of residual disease or disease recurrence in the patients within these categories on follow-up. The results of this study cannot accurately determine the risk of residual disease and the ACPGBI risk scoring tool’s accuracy for these patients due to the extremely small number of patients who fell within these categories (n = 1 for ACPGBI risk scores 2 and 3 combined).

The management recommendations from the ACPGBI risk scoring system are appropriate for patients scoring 0, 1, and 4 or more. However, the estimated potential percentage of risk of residual cancer needs further assessment and adjustment. Overestimating the potential risk of residual cancer indicates patients are at risk of undergoing unnecessary surgical intervention, which can have life-changing sequelae, while underestimating the potential risk may result in definitive and curative management delay in patients and therefore risk progression of disease both locally and systemically.

A secondary outcome of this study was to examine other potential characteristics that can predict the risk of residual disease. Polyp size was shown to be significant across the total patient population and specifically in those that had surgery within the high-risk category (score ≥4). Half of the patients with a polyp larger than 40 mm had residual disease, compared to none with polyps less than 10 mm in this study. Barosa et al. [11] report that polyps of 32 mm or larger were statistically more likely to predict higher rates of residual disease at the polypectomy site. Moss et al. [12] also recognized that lesions >30 mm carried a higher risk of early recurrence after endoscopic mucosal resection (EMR), with lesions >40 mm having an OR of 8.22 [12]. Further assessment of this variable is warranted, as it is not currently used in the ACPGBI tool and may help improve overall accuracy.

Further research into the accuracy of the ACPGBI risk scoring system is required, particularly focusing on the percentage risk of residual cancer in a multi-center study with a larger population over a follow-up period of more than one year. Limitations of this study include a small sample size in a single-center study, alongside a short follow-up period of only one year. Despite these limitations, however, our research supports the values reported within the tool itself, proving it is useful and safe. Improvement in the tool through further research and continued use in practice will result in an accurate and invaluable tool for all clinicians involved in the management of malignant colorectal polyps.

## Conclusions

The ACPGBI risk assessment tool is accurate in predicting the risk of residual disease in endoscopically resected malignant polyps. It should be consistently used in MDT discussions to aid clinicians in decision-making. More research investigating risk factors that predict the risk of residual disease, such as polyp size, is warranted to improve the risk tool’s accuracy. The ongoing development of this risk assessment tool will create an invaluable resource for clinicians in the future management of malignant colorectal polyps.
